# Medication Adherence in Elderly Diabetic Patients: A Cross-Sectional Study From Dakshina Kannada, India

**DOI:** 10.7759/cureus.43098

**Published:** 2023-08-07

**Authors:** Hrushikesh Udupa, Anusree Viswanath, Pooja Umesh Shenoy, Karen Jennifer Antao, Ranajit Das

**Affiliations:** 1 Community Medicine, Yenepoya Medical College, Yenepoya (Deemed to be University), Mangalore, IND; 2 Data Analytics, Bioinformatics and Structural Biology (DABS), Yenepoya Research Centre, Yenepoya (Deemed to be University), Mangalore, IND

**Keywords:** diabetic patient, geriatric patient, medication adherence rating scale, medications adherence, diabete mellitus

## Abstract

Diabetes Mellitus (DM) has emerged as a major global healthcare problem. The risk of diabetes can be reduced by maintaining blood glycaemic levels, which can be achieved by stringent adherence to the treatment regime. Therefore, there is a continuing need to assess the level of adherence to medication/self-care activities and the factors that are related to non-adherence to medication and self-care. This would facilitate healthcare professionals to identify subjects with low medication adherence and thereby aid them in planning interventions to improve medication and self-care adherence. In this study, we aimed to estimate the proportion of medication adherence among diabetic patients above 60 years of age attending a tertiary care hospital in Southern India. We found that 72% of type 2 diabetes patients were adherent to the medications prescribed to them and there was a discernible effect of gender and literacy on medication adherence. However, more such regional studies need to be conducted with a larger sample size from diverse hospital setups to obtain a clear and unbiased picture of the drug adherence scenario in India.

## Introduction

Diabetes mellitus (DM) is a chronic, metabolic disease characterized by elevated levels of blood glucose, which over time can lead to serious damage to the heart, blood vessels, eyes, kidneys, and nerves. About 422 million people worldwide have diabetes, with the majority living in low-and middle-income countries, and 1.5 million deaths are directly attributed to diabetes each year [1]. Diabetes mellitus has emerged as a major healthcare problem in India*.*

According to the World Health Organization, treatment adherence is " the extent to which a person's behaviour - taking medication, following a diet, and/or executing lifestyle changes - corresponds with the agreed recommendations from a healthcare provider [2]." Nonadherence to medication declines the efficacy of the medication and, in turn, glycaemic control. There is also a continuing need to assess the level of adherence to medication and the factors related to non-adherence to medication and self-care among diabetics in the local setting. This would facilitate healthcare professionals to identify subjects with low medication adherence, thereby aiding them in planning interventions to improve medication and self-care adherence.

The Alberta’s Tomorrow Project is a long-term, population-based research project that aims to investigate the determinants of chronic diseases, including diabetes, in Alberta, Canada. A study conducted using data from this project examined the association between anti-hyperglycemic medication adherence and healthcare utilization in people with diabetes over a four-year period [3]. The study found that non-adherent individuals had significantly higher rates of emergency department visits, hospitalizations, and physician visits than adherent individuals. Non-adherent individuals were also more likely to have uncontrolled diabetes, as indicated by higher glycated haemoglobin (HbA1c) levels and were less likely to achieve recommended cholesterol and blood pressure targets [3].

Previous studies have also reported similar findings, highlighting the importance of medication adherence in diabetes management and reducing healthcare utilization. A systematic review and meta-analysis of 25 studies found that medication adherence was associated with a 0.65-fold decrease in the risk of hospitalizations and a 0.75-fold decrease in emergency department visits [4].

Several factors can influence medication adherence in people with diabetes, including socioeconomic status, education, health literacy, medication complexity, and side effects. Strategies to improve medication adherence in diabetes management include patient education, simplifying medication regimens, using reminder systems, and involving patients in shared decision-making with healthcare providers [5]. It has been shown that increasing medication adherence among type 2 diabetes mellitus (T2DM) patients can aid in HbA1c control [6]. Another study by Hussain-Gambles et al. (2004) explored the experiences of patients with T2DM who were non-adherent to their medications. The study found that factors such as forgetfulness, competing priorities, and negative perceptions of medication were common reasons for non-adherence. The study also found that patients who were non-adherent to their medications were more likely to have poor glycemic control [7].

A recent study investigated the impact of the COVID-19 pandemic on medication adherence and healthcare use among high-risk patients with diabetes. The study found that medication adherence decreased during the pandemic, and there was a decrease in healthcare utilization, including fewer in-person visits and laboratory testing. These findings suggest that the COVID-19 pandemic may have had a negative impact on the management of diabetes [8]. Several studies have investigated medication adherence and healthcare utilization in patients with diabetes before the COVID-19 pandemic. Qiao et al. (2020) found that poor medication adherence was associated with higher healthcare costs among patients with diabetes [9]. The use of telehealth may be a potential solution to the challenges posed by the COVID-19 pandemic in the management of diabetes. A recent study has found that telehealth was associated with improved medication adherence and glycemic control in patients with diabetes [10]. Mobile health (mHealth) interventions may be a potential solution to the challenges posed by poor medication adherence in patients with diabetes. A recent study investigated the effectiveness of a mHealth intervention on medication adherence in patients with T2DM. The study found that the intervention effectively improved medication adherence and glycemic control in patients with poor adherence at baseline [11].

A systematic review identified several factors associated with poor medication adherence in patients with diabetes. These factors included poor knowledge of diabetes and its treatment, poor social support, medication side effects, and poor provider-patient communication [12]. Medical adherence is a crucial factor in the effective management of diabetes. Adherence to medications, lifestyle modifications, and self-care behaviours can reduce the risk of complications and improve overall glycemic control [13]. However, several studies have reported low levels of adherence among individuals with diabetes, especially from low-income countries. A study set in Ghana revealed adherence to oral hypoglycemic drugs among T2DM patients to be 47.75% [14]. Another study conducted in Pakistan found that 55.6% of patients with T2DM were adherent to their medications [15]. These low adherence rates have been linked to increased healthcare costs, complications, and mortality.

The prevalence of diabetes in India is high, making it a major cause of morbidity and mortality. To mitigate the risk of complications associated with diabetes, maintaining good glycemic control is crucial. However, non-adherence to medication and self-care activities is a common issue. Identifying factors contributing to non-adherence and planning interventions to improve adherence can help healthcare professionals manage diabetes better and improve the quality of life for those living with the condition. In this study, we aimed to estimate the proportion of medication adherence among diabetic patients above 60 years attending a tertiary hospital and identify the factors affecting medication adherence in elderly patients with diabetes mellitus.

## Materials and methods

Study setting

This study was carried out in the outpatient department (OPD) of Yenepoya Medical College Hospital (YMCH), a tertiary care hospital in Mangalore city, India, for a period of 5 months from April 2022 to August 2022. Elderly individuals (>60 years old) who were previously diagnosed with T2DM and were on antidiabetic medication for at least 1 month and attending the OPD in YMCH during the aforementioned period were included in the study.

Method of data collection 

A hospital-based cross-sectional study was conducted upon obtaining clearance from the Institutional Ethics Committee (Yenepoya Ethics Committee-1 ) with approval number YEC-1/2022/058. The interview included a pre-designed, pre-tested, structured questionnaire. Information regarding patient characteristics, diabetes medication adherence, adherence to self-care measures, and family support were collected. Medication adherence was measured by using Medication Adherence Rating Scale (MARS). The adherence was classified as Non-Adherent (0-3), Partially Adherent (4-6), and Adherent (7-10) [16]**.**

Details of statistical analysis

The outcomes of questionnaire responses were recorded on a Microsoft Excel sheet (Microsoft Corporation, Redmond, USA). Data were further compiled and analyzed using the Statistical Package for Social Sciences (SPSS), Version 23 (IBM Corp., Armonk, USA). Continuous variables were expressed in terms of means and medians. Proportions and percentages were used to express categorical variables.

## Results

The study involved 101 participants with a mean age (±SD) of 66.14 ± 5.81 years. The demographic variables are summarised in Table 1.

**Table 1:** Socio-demographic characteristics of the participants in the study (n=101)

Variables	Category	Number (n)	Percentage (%)
Age groups (years)	60-65	58	57.4
	66-70	20	19.8
	71-75	14	13.9
	76-80	9	8.9
Gender	Male	72	71.3
	Female	29	28.7
Religion	Hindu	37	36.6
	Muslim	60	59.4
	Christian	4	4
Socioeconomic class (Modified BG Prasad Classification [14])	Class I	9	8.9
	Class II	10	9.9
	Class III	18	17.8
	Class IV	25	24.8
	Class V	39	38.8
Education	Illiterate	26	25.7
	Primary school	43	42.6
	Middle school	15	14.9
	High school	6	5.9
	Intermediate and above	11	10.9
Occupation	Salaried person	9	8.9
	Self-employed	48	47.5
	Homemaker	24	23.8
	Unemployed	13	12.9
	Retired	7	6.9

While 42.6% of the male participants had primary school education, nearly 1/3rd of the women participants did not attend school or had unknown educational status. Among the study participants, the majority (39%) belonged to socio-economic class V with a per capita income below Rs 1183 per month based on the Modified BG Prasad Classification [17]. Nearly 81% were on oral medication for T2DM, and among whom, 51% were on a single medication. The median adherence score was 3. The percentage of participants taking insulin for diabetes and suffering from other co-morbid conditions requiring prolonged treatment was 37% and 48 %, respectively.

Only ~27% of participants said they forgot to take the medication. Interestingly, out of 29 female participants, 24 claimed (83%) that they never forget to take their medication; a discernibly lower number (69%) of male participants claimed the same. The majority of the participants (82%) claimed they are never careless about taking their medication. There was no discernible gender difference for this claim (81% males vs. 79% females). Only 22% of participants claimed they sometimes stop taking their medication if they feel better. 87% of participants, without any discernible gender variation, claimed that they do not stop taking the medication even when they feel worse after taking it. 86% of the participants adhered to their medications regardless of whether they were sick.

**Figure 1:**
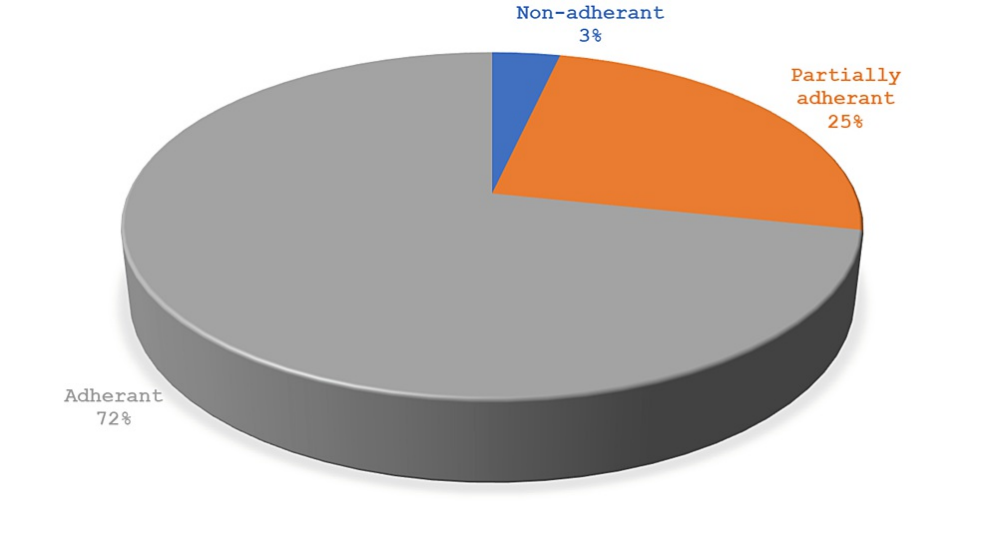
Adherence of the participants to their medication

Overall, based on MARS, 72% of the participants were adherent to the therapeutic regime (Figure 1)

**Figure 2:**
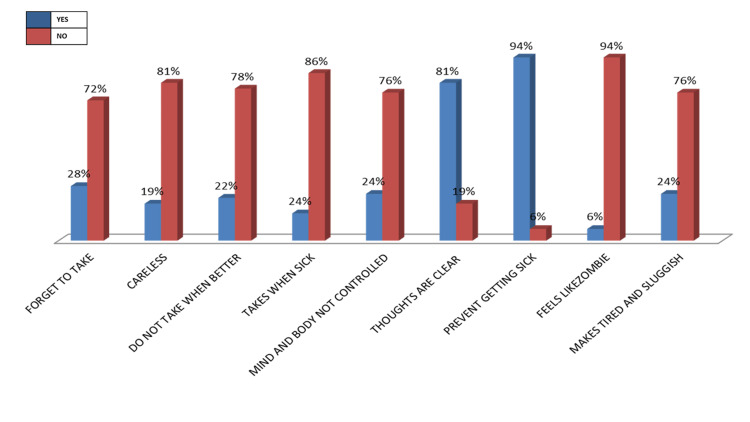
Response of the participants to the adherence rating questionnaire

The majority of the participants (94%) believe that adhering to the medication can prevent them from getting sick, indicating their trust in the given medication. While only 6% of participants feel weird, like a ‘zombie’ on medication, 24% said the medication made them feel tired and sluggish (Figure 2).

## Discussion

The current study assessed medication adherence among T2DM patients aged above 60 years attending a tertiary care hospital in Dakshina Kannada district of Karnataka, India. We found that 72% of T2DM patients adhered to the prescribed medications, which was notably higher than in a similar study conducted in India, where 49.3% of diabetes patients exhibited medication adherence [18] as well as in our neighboring country [15]. This could be attributed to the fact that the majority were on single oral medication and the availability of several healthcare facilities in the study area, and good healthcare utilization among the study participants. However, the medication adherence rate in our study group was slightly lower than that reported by Karter et al., who showed 22.3% of the participants did not continue to take the new prescription, indicating a medication adherence of ~78% [19]. Moreover, another study found that insulin adherence was 62-64% in T2DM patients, which is slightly lower than our results [ 20].

The aforementioned study by S. Arulmozhi and T. Mahalakshmi found that medical adherence was nearly eighty per cent (79.5%) among the literates, very similar to what we observed in our study regarding the medical adherence among literates (~80%) [18].

According to Donnan et al., a key impediment to the benefits of complex medication regimens in treating T2DM is poor adherence [21], similar to the findings of our study showing higher adherence among those who were on single oral medication for DM. Jankowska-Polańska B et al. recently showed that the level of adherence was highest in patients with diabetes alone and was lowest in patients with co-existing hypertension and diabetes [22]. A similar finding was observed in this study, where 48% had some chronic illness requiring prolonged medication. This indicates that prescribing multiple drugs alongside T2DM medications may lead to poor medication adherence.

The current study was conducted in one tertiary care hospital, which may pose a significant limitation to this study regarding the diversity of the participants.

## Conclusions

Overall, our study is one of the handful of studies in India that assesses medication adherence among patients. Our results are encouraging, indicating a medication adherence rate of over 70% for T2DM patients. We also observed a discernible effect of gender and education on drug adherence, with females and educated individuals demonstrating higher adherence to their medications compared to men and those with lower education.

More such studies should be conducted with a larger sample size from diverse hospital setups to obtain a clear and unbiased picture of the drug adherence scenario in India.
